# From Access to Watch: An AWaRe-Oriented Evaluation of Antibiotic Prescribing in ASL Salerno

**DOI:** 10.3390/antibiotics15060612

**Published:** 2026-06-17

**Authors:** Angelo Santoro, Rosaria Toro, Sergio Esposito, Antonio Lalli, Aniello Corallo, Francesca Futura Bernardi, Gennaro Sosto, Mariarosaria Cillo, Anna Maria D’Ursi

**Affiliations:** 1 Department of Pharmacy, University of Salerno, 84084 Salerno, Italy; asantoro@unisa.it; 2 Scuola di Specializzazione in Farmacia Ospedaliera, Department of Pharmacy, University of Salerno, 84084 Salerno, Italy; 3 Dipartimento Farmaceutico, Local Health Authority (ASL) of Salerno, 84124 Salerno, Italy; r.toro@aslsalerno.it (R.T.); s.esposito@aslsalerno.it (S.E.); m.cillo@aslsalerno.it (M.C.); 4 Fatebenefratelli Hospital, SIFO (“Società Italiana di Farmacia Ospedaliera e Dei Servizi Farmaceutici Delle Aziende Sanitarie”) Campania Section, 80100 Naples, Italy; lalli.antonio@fbfna.it; 5 Local Health Authority (ASL) of Salerno, SIFO (“Società Italiana di Farmacia Ospedaliera e dei Servizi Farmaceutici delle Aziende Sanitarie”) Campania Section, 84131 Salerno, Italy; a.corallo@aslsalerno.it; 6 General Directorate for Health Protection, SIFO (“Società Italiana di Farmacia Ospedaliera e Dei Servizi Farmaceutici Delle Aziende Sanitarie”) Campania Section, 80100 Naples, Italy; francescafutura.bernardi@regione.campania.it; 7 General Directorate, Local Health Authority (ASL) of Salerno, 84134 Salerno, Italy; direzionegenerale@aslsalerno.it

**Keywords:** antimicrobial stewardship, AWaRe classification, antibiotics, antimicrobial resistance, pharmacoepidemiology

## Abstract

Background/Objectives: The present study aimed to analyze systemic antibiotic prescription patterns in the Salerno Local Health Authority (ASL Salerno) during 2024. The objective was to describe territorial antibiotic consumption and monitor prescribing appropriateness from epidemiological, pharmacological, and antimicrobial stewardship perspectives, using the World Health Organization (WHO) AWaRe classification system as a reference. Methods: A retrospective analysis of antibiotic prescriptions was conducted across ASL Salerno in 2024. Total prescription and associated expenditures were assessed, along with distribution across districts and pharmacological classes. Antibiotics were categorized according to the Anatomical Therapeutic Chemical (ATC) system and further classified using the WHO AWaRe framework (Access, Watch, Reserve) to evaluate prescribing appropriateness and potential patterns of antimicrobial resistance. Results: More than 1 million antibiotic prescriptions were recorded, totaling approximately €15 million in expenditure. Prescribing patterns showed a predominance of penicillins and cephalosporins, followed by macrolides, fluoroquinolones, and J01X class. A substantial proportion of prescriptions belonged to the Watch category, particularly third-generation cephalosporins, fluoroquinolones, and fosfomycin. Some districts demonstrated a higher reliance on Watch antibiotics, while others showed increased use of Access agents such as amoxicillin, doxycycline, and amikacin. Conclusions: The surveillance analysis highlights significant variability in antibiotic prescribing practices within ASL Salerno and a substantial use of Watch-group antibiotics. These findings suggest the need for strengthened antimicrobial stewardship interventions, such as targeted education, auditing, and decision-support tools. Continuous monitoring of prescribing trends is essential to encourage appropriate antibiotic use and contribute to the containment of antimicrobial resistance.

## 1. Introduction

Antimicrobial resistance (AMR) has emerged as one of the most demanding public health challenges of the 21st century, posing a substantial threat to modern medicine [1]. Indeed, the increasing incidence of resistant microorganisms undermines the efficacy of antibiotics and exposes decades of progress in infectious disease control. The inappropriate and excessive use of antibiotics is recognized as one of the main drivers of AMR, leading to prolonged hospitalizations, higher healthcare costs, treatment failures, and, finally, increased mortality. In response to this global health crisis, the World Health Organization (WHO) has established several regulatory frameworks and strategic guidelines to promote the prudent use of antimicrobials. Among these, the AWaRe classification system represents a key tool for antibiotic management [2,3]. In particular, the AWaRe framework, an acronym for Access, Watch, and Reserve, was developed by WHO to assist countries in monitoring antibiotic consumption and promoting rational prescribing practices [4]. This categorization classifies antibiotics according to their therapeutic importance, resistance potential, and public health relevance. The “Access” group includes antibiotics that should be widely available, affordable, and associated with lower resistance risk; the “Watch” group includes molecules with higher resistance potential and more restricted indications; and the “Reserve” group encompasses last-resort antibiotics for multidrug-resistant infections [5]. By promoting preferential use of Access antibiotics and decreasing the inappropriate use of Watch and Reserve agents, AWaRe serves as an operational framework to harmonize global efforts in AMR management [6]. One of the most widely adopted systems for quantifying drug utilization is the Defined Daily Dose (DDD) [7]. When integrated with the AWaRe framework, this metric allows assessment not only of the volume but also of the appropriateness of antibiotic use. This synergy enables a more detailed analysis of prescribing trends, highlighting deviations from recommended practices and identifying areas requiring stewardship interventions [8].

**Figure 1:**
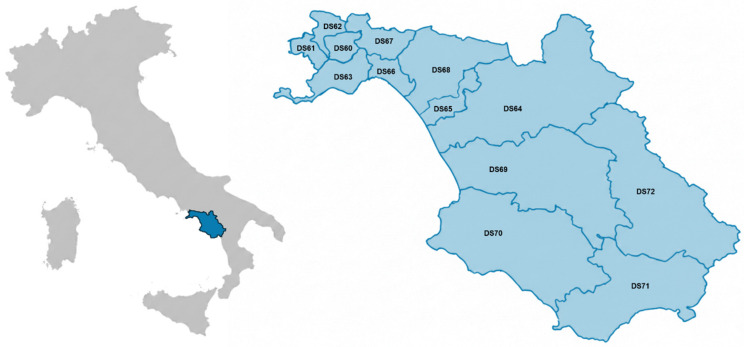
Map of ASL Salerno showing the subdivision into health districts and their geographic coverage: DS60 (Nocera Inferiore), DS61 (Angri-Scafati), DS62 (Sarno-Pagani), DS63 (Cava de’ Tirreni-Costa d’Amalfi), DS64 (Eboli-Buccino), DS65 (Battipaglia), DS66 (Salerno city), DS67 (Mercato San Severino), DS68 (Giffoni Valle Piana), DS69 (Capaccio-Roccadaspide), DS70 (Vallo della Lucania-Agropoli), DS71 (Sapri), and DS72 (Sala Consilina-Polla).

In 2023, antibiotic consumption in Europe averaged around 20 DDD per 1000 inhabitants per day (DDD/1000 ab/die). In terms of consumption quality, the Access group represented approximately 61.5% of the total [9]. Although this value has improved compared to previous years, it remains below the WHO and European Union target of at least 65% by 2030 [10]. Nevertheless, surveillance data from various healthcare contexts reveal a disproportionate reliance on Watch and Reserve antibiotics, reflecting gaps in guideline adherence, diagnostic uncertainty, and variability in prescriber behavior. National AMR action plans, though increasingly common, often lack the granularity needed to address context-specific challenges within healthcare systems. Italy exemplifies this issue, as it continues to report antibiotic consumption levels above the European average. According to the most recent AIFA-OsMed report, in 2023 consumption reached 23.1 DDD per 1000 inhabitants per day, a 5.5% increase compared with the previous year. The AWaRe distribution is even more concerning: only 50.8% of antibiotics prescribed within the National Health Service were in the Access group, a significantly lower proportion than recommended, suggesting a persistently excessive use on Watch and Reserve antibiotics. Considerable regional variation further describes the national landscape [9]. Southern regions, including Campania, consistently report higher consumption rates. A particularly relevant case is represented by ASL Salerno. This ASL is among the largest and most complex in Italy, both in terms of territorial extension and population served. Its jurisdiction encompasses 13 healthcare districts (DS60-DS72), covering approximately 4954 km^2^ and serving a total population of 1,054,766 inhabitants. The population is distributed between densely populated urban areas and predominantly rural or mountainous territories, leading to the functional subdivision of the ASL into three macro-areas: north, center, and south (Figure 1).

The northern area, including DS60 (Nocera Inferiore, 77,007 inhabitants), DS61 (Angri-Scafati, 84,702 inhabitants), DS62 (Sarno-Pagani, 79,248 inhabitants), and DS63 (Cava de’ Tirreni-Costa d’Amalfi, 86,726 inhabitants), is characterized by high urbanization and population density. Major urban centers, high-volume hospitals, and substantial pressure on primary care are concentrated here. Closeness between municipalities, better-developed transportation networks, and greater accessibility to healthcare services represent both strengths and managerial challenges. The central area, comprising DS64 (Eboli-Buccino, 110,100 inhabitants), DS65 (Battipaglia, 69,302 inhabitants), DS66 (Salerno, 136,842 inhabitants), DS67 (Mercato San Severino, 68,388 inhabitants), and DS68 (Giffoni Valle Piana, 68,880 inhabitants), represents a geographical and functional transition zone. It includes medium-sized urban poles alongside more remote hilly or inland areas. The variability of these contexts translates into heterogeneous healthcare needs and organizational models. Salerno (DS66) in particular acts as a provincial healthcare hub, hosting major hospital facilities and a high concentration of specialists.

Nevertheless, the complexity of the catchment population results in service overload and coordination challenges across levels of care. The southern area, consisting of DS69 (Capaccio-Roccadaspide, 62,430 inhabitants), DS70 (Vallo della Lucania-Agropoli, 99,497 inhabitants), DS71 (Sapri-Camerota, 43,126 inhabitants), and DS72 (Sala Consilina-Polla, 68,518 inhabitants), covers a vast, predominantly hilly and mountainous territory with small, scattered municipalities. Low population density, geographic dispersion, and an older demographic profile make this area particularly vulnerable to healthcare accessibility, diagnostic timeliness, and continuity of care. Logistical issues, shortages of medical personnel, and the need for integrated care pathways represent the main challenges, highlighting the importance of strengthening community-based care and home services. The district-based organization of ASL Salerno reflects an attempt to adapt territorial healthcare to the province’s complexity, promoting governance strategies that address local specificities. However, the differences between the northern, central, and southern areas underscore the need for differentiated health policies tailored to community-specific needs regarding equitable access to care and appropriate prescribing. This study aims to conduct a detailed surveillance analysis of antibiotic prescriptions within ASL Salerno in 2024, leveraging administrative data to extract DDD metrics and map antibiotic use according to the AWaRe classification. The objectives are to determine the relative proportion of antibiotics prescribed across Access, Watch, and Reserve groups, identify the most commonly used molecules within each category, and evaluate adherence to WHO recommendations. By integrating consumption data within the AWaRe taxonomy, this research provides insights to improve local antimicrobial stewardship strategies. This surveillance analysis is expected to inform both institutional policies and broader regional efforts to align antibiotic use with global standards. Moreover, this study contributes to the growing body of literature underscoring the importance of integrating investigation data with stewardship frameworks to address AMR effectively. Ultimately, this investigation offers a replicable model for other healthcare systems facing similar challenges. Given the relentless emergence of resistant pathogens and limited therapeutic options, optimizing antibiotic use through evidence-based frameworks such as AWaRe is not merely an academic endeavor but an imperative for safeguarding public health.

## 2. Results

**Figure 2:**
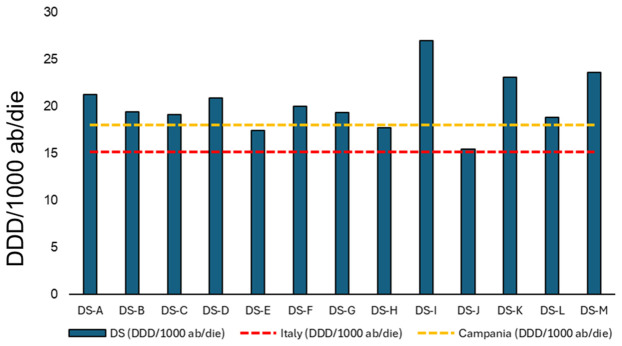
DDD of antibiotics per 1000 inhabitants per day across the analyzed districts (DS-A to DS-M). The red dashed line indicates the Italian national value, while the yellow dashed line represents the Campania regional value.

In 2024, more than one million systemic antibiotic prescriptions were issued within ASL Salerno, amounting to approximately €14.9 million in expenditure. The impact of this therapeutic class on territorial pharmaceutical spending is particularly relevant. It warrants in-depth analysis, both quantitative and qualitative, to identify potential criticalities and guide strategies to promote appropriate prescribing. To further contextualize antibiotic utilization within the ASL, overall outpatient prescription care consumption was evaluated using DDD/1000 ab/die and compared with both regional (Campania) and national Italian reference values. The average antibiotic consumption across the districts was consistently higher than the national benchmark (15.10 DDD/1000 ab/die) and exceeded the regional average for Campania (17.98 DDD/1000 ab/die) in most districts. Considerable inter-district variability emerged. The highest consumption values were observed in DS-I (27.02 DDD/1000 ab/die), DS-M (23.63), and DS-K (23.08), indicating substantially greater exposure to systemic antibiotics than in both regional and national references. By contrast, DS-J showed the lowest consumption level (15.44 DDD/1000 ab/die), closely approaching the Italian average, while DS-E (17.44) and DS-H (17.72) remained slightly below the regional benchmark (Figure 2).

**Figure 3:**
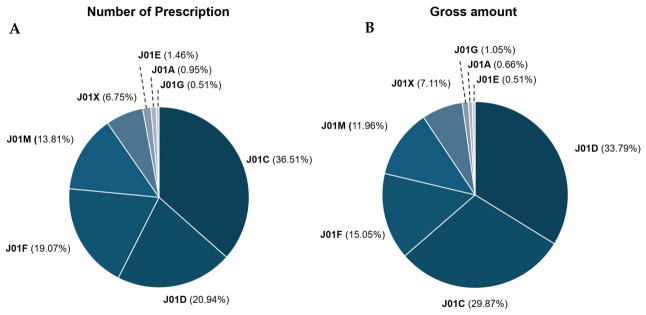
Pie charts illustrating antibiotic consumption across the 13 health districts of ASL Salerno, expressed as (**A**) number of prescriptions and (**B**) gross expenditure amount, by antibiotic class. Antibiotic classes are defined according to ATC codes: J01 (tetracyclines), J01C (penicillins), J01D (cephalosporins and other beta-lactam antibiotics), J01F (macrolides, lincosamides, and streptogramins), J01G (aminoglycosides), J01M (fluoroquinolones), and J01X (other antibiotics).

At the overall level, prescriptions were concentrated mainly in four large antibiotic classes: penicillins (J01C), accounting for more than one-third of prescriptions and nearly 30% of expenditure; cephalosporins and other beta-lactams (J01D), which represented just over 20% of prescriptions but absorbed more than one-third of total costs; macrolides and lincosamides (J01F), comprising around 19% of prescriptions and 15% of expenditure; and fluoroquinolones (J01M), responsible for approximately 14% of prescriptions and 12% of spending. The remaining classes, such as tetracyclines, sulfonamides, aminoglycosides, and specialized-use antibiotics, had a marginal weight in both prescriptions and expenditure, but deserve specific attention, particularly for their potential role in selective and responsible antibiotic use (Figure 3).

**Figure 4:**
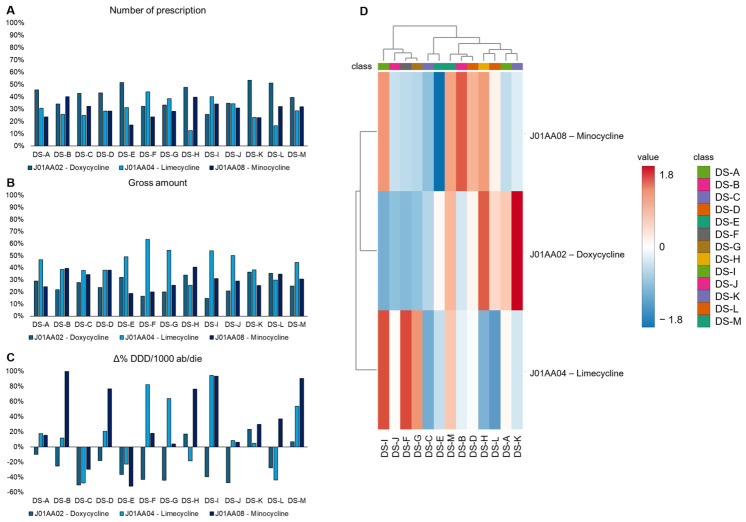
Percentage distribution by district of (**A**) prescriptions and (**B**) gross expenditure for J01A antibiotics. (**C**) Percentage variation (Δ%) in DDD/1000 inhabitants/day relative to the Italian national benchmark for each antibiotic class across districts; positive values indicate consumption above the national benchmark and negative values below it. (**D**) Hierarchical clustering heatmap integrating prescription frequency, gross expenditure, and Δ% DDD/1000 inhabitants/day across the 13 health districts. The vertical dendrogram (**top**) groups districts based on the similarity of their overall prescribing profiles, while the horizontal dendrogram (**left**) groups active substances by their distribution patterns. The color scale represents Z-score standardized values, with a gradient transitioning from dark blue (lower consumption/spending or negative variation relative to the national benchmark) to intense red (higher consumption/spending or positive variation above the benchmark). White/light cells represent values near the sample mean.

**Figure 5:**
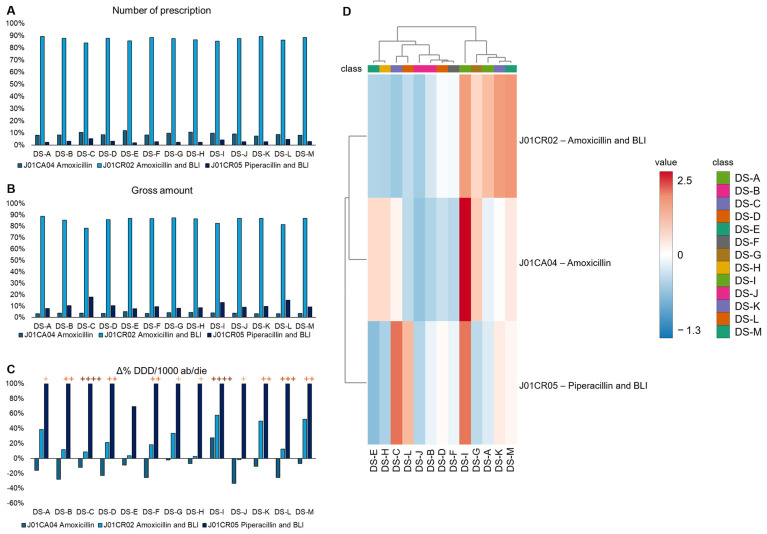
Percentage distribution by district of (**A**) prescriptions and (**B**) gross expenditure for J01C antibiotics. (**C**) Percentage variation (Δ%) in DDD/1000 inhabitants/day relative to the Italian national benchmark for each antibiotic class across districts, positive values indicate consumption above the national benchmark and negative values below it. To improve graphical readability, extreme positive Δ% values were annotated as follows: +, 100% < Δ% < 200%; ++, 200% < Δ% < 300%; +++, 300% < Δ% < 400%; ++++, 400% < Δ% < 500%. (**D**) Hierarchical clustering heatmap integrating prescription frequency, gross expenditure, and Δ% DDD/1000 inhabitants/day across the 13 health districts. The vertical dendrogram (**top**) groups districts based on the similarity of their overall prescribing profiles, while the horizontal dendrogram (**left**) groups active substances by their distribution patterns. The color scale represents Z-score standardized values, with a gradient transitioning from dark blue (lower consumption/spending or negative variation relative to the national benchmark) to intense red (higher consumption/spending or positive variation above the benchmark). White/light cells represent values near the sample mean.

**Figure 6:**
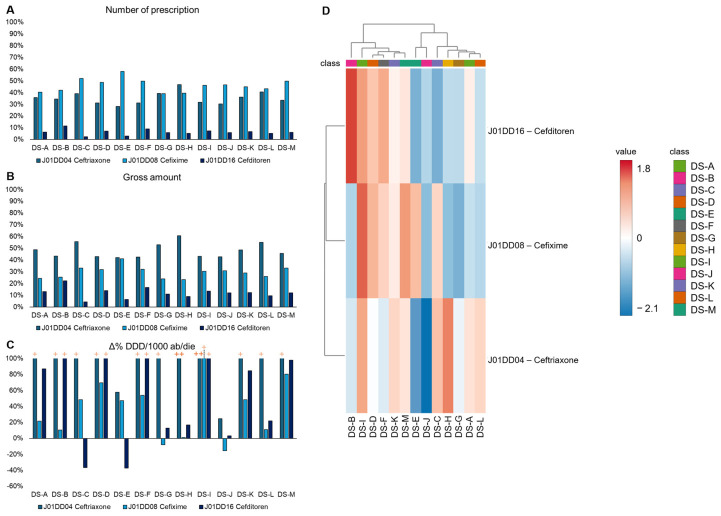
Percentage distribution by district of (**A**) prescriptions and (**B**) gross expenditure for J01D antibiotics. (**C**) Percentage variation (Δ%) in DDD/1000 inhabitants/day relative to the Italian national benchmark for each antibiotic class across districts; positive values indicate consumption above the national benchmark and negative values below it. To improve graphical readability, extreme positive Δ% values were annotated as follows: +, 100% < Δ% < 200%; ++, 200% < Δ% < 300. (**D**) Hierarchical clustering heatmap integrating prescription frequency, gross expenditure, and Δ% DDD/1000 inhabitants/day across the 13 health districts. The vertical dendrogram (**top**) groups districts based on the similarity of their overall prescribing profiles, while the horizontal dendrogram (**left**) groups active substances by their distribution patterns. The color scale represents Z-score standardized values, with a gradient transitioning from dark blue (lower consumption/spending or negative variation relative to the national benchmark) to intense red (higher consumption/spending or positive variation above the benchmark). White/light cells represent values near the sample mean.

**Figure 7:**
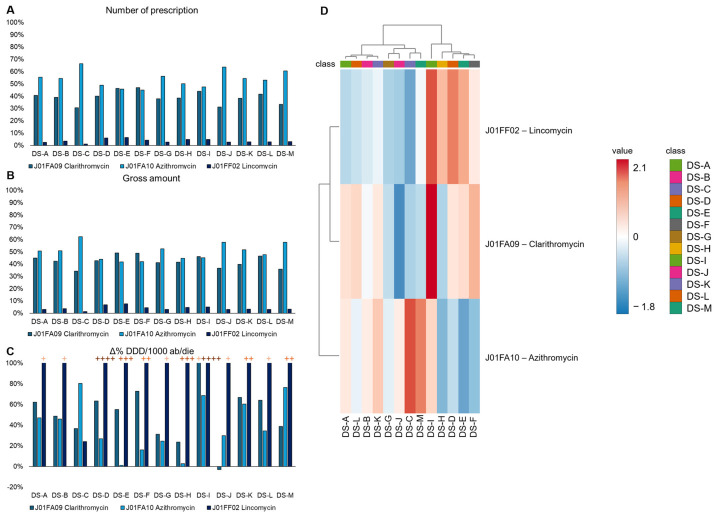
Percentage distribution by district of (**A**) prescriptions and (**B**) gross expenditure for J01F antibiotics. (**C**) Percentage variation (Δ%) in DDD/1000 inhabitants/day relative to the Italian national benchmark for each antibiotic class across districts; positive values indicate consumption above the national benchmark and negative values below it. To improve graphical readability, extreme positive Δ% values were annotated as follows: +, 100% < Δ% < 200%; ++, 200% < Δ% < 300%; +++, 300% < Δ% < 400%; ++++, 400% < Δ% < 500%. (**D**) Hierarchical clustering heatmap integrating prescription frequency, gross expenditure, and Δ% DDD/1000 inhabitants/day across the 13 health districts. The vertical dendrogram (**top**) groups districts based on the similarity of their overall prescribing profiles, while the horizontal dendrogram (**left**) groups active substances by their distribution patterns. The color scale represents Z-score standardized values, with a gradient transitioning from dark blue (lower consumption/spending or negative variation relative to the national benchmark) to intense red (higher consumption/spending or positive variation above the benchmark). White/light cells represent values near the sample mean.

**Figure 8:**
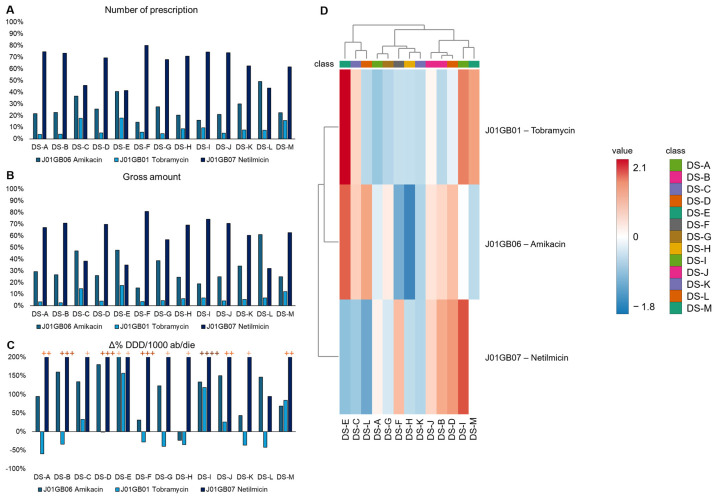
Percentage distribution by district of (**A**) prescriptions and (**B**) gross expenditure for J01G antibiotics. (**C**) Percentage variation (Δ%) in DDD/1000 inhabitants/day relative to the Italian national benchmark for each antibiotic class across districts; positive values indicate consumption above the national benchmark and negative values below it. To improve graphical readability, extreme posi-tive Δ% values were annotated as follows: +, 200% < Δ% < 500%; ++, 500% < Δ% < 800%; +++, 800% < Δ% < 1100%; ++++, 1100% < Δ% < 1400%. (**D**) Hierarchical clustering heatmap integrating prescription frequency, gross expenditure, and Δ% DDD/1000 inhabitants/day across the 13 health districts. The vertical dendrogram (**top**) groups districts based on the similarity of their overall prescribing profiles, while the horizontal dendrogram (**left**) groups active substances by their distribution patterns. The color scale represents Z-score standardized values, with a gradient transitioning from dark blue (lower consumption/spending or negative variation relative to the national benchmark) to intense red (higher consumption/spending or positive variation above the benchmark). White/light cells represent values near the sample mean.

**Figure 9:**
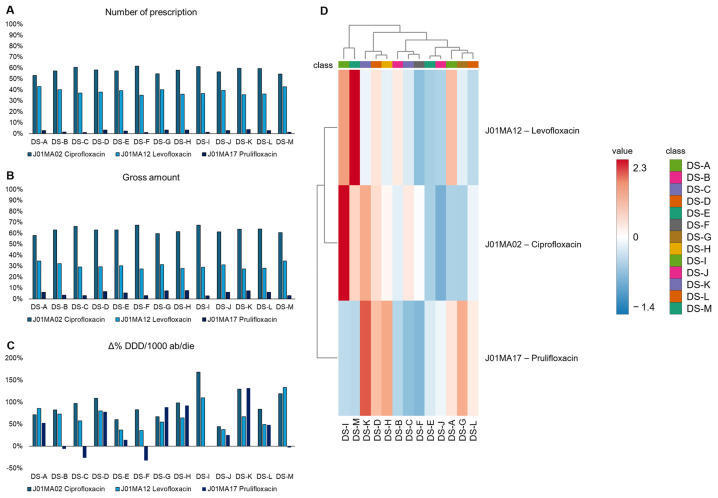
Percentage distribution by district of (**A**) prescriptions and (**B**) gross expenditure for J01M antibiotics. (**C**) Percentage variation (Δ%) in DDD/1000 inhabitants/day relative to the Italian national benchmark for each antibiotic class across districts; positive values indicate consumption above the national benchmark and negative values below it. (**D**) Hierarchical clustering heatmap integrating prescription frequency, gross expenditure, and Δ% DDD/1000 inhabitants/day across the 13 health districts. The vertical dendrogram (**top**) groups districts based on the similarity of their overall prescribing profiles, while the horizontal dendrogram (**left**) groups active substances by their distribution patterns. The color scale represents Z-score standardized values, with a gradient transitioning from dark blue (lower consumption/spending or negative variation relative to the national benchmark) to intense red (higher consumption/spending or positive variation above the benchmark). White/light cells represent values near the sample mean.

**Figure 10:**
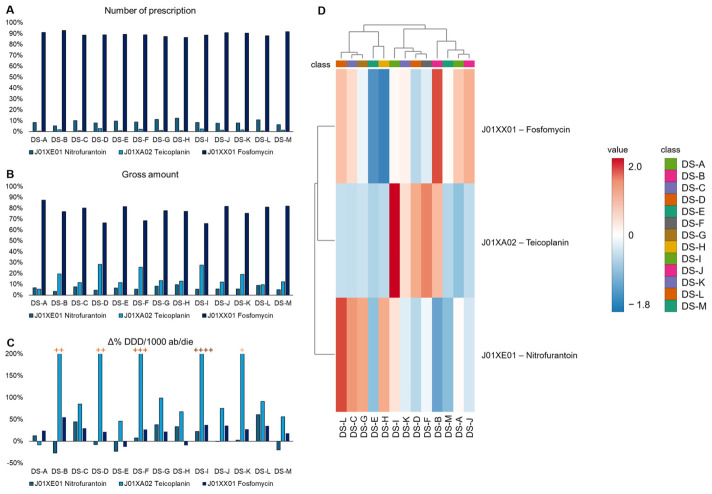
Percentage distribution by district of (**A**) prescriptions and (**B**) gross expenditure for J01X antibiotics. (**C**) Percentage variation (Δ%) in DDD/1000 inhabitants/day relative to the Italian national benchmark for each antibiotic class across districts; positive values indicate consumption above the national benchmark and negative values below it. To improve graphical readability, extreme posi-tive Δ% values were annotated as follows: +, 200% < Δ% < 300%; ++, 300% < Δ% < 400%; +++, 400% < Δ% < 500%; ++++, 500% < Δ% < 600%. (**D**) Hierarchical clustering heatmap integrating prescription frequency, gross expenditure, and Δ% DDD/1000 inhabitants/day across the 13 health districts. The vertical dendrogram (**top**) groups districts based on the similarity of their overall prescribing profiles, while the horizontal dendrogram (**left**) groups active substances by their distribution patterns. The color scale represents Z-score standardized values, with a gradient transitioning from dark blue (lower consumption/spending or negative variation relative to the national benchmark) to intense red (higher consumption/spending or positive variation above the benchmark). White/light cells represent values near the sample mean.

Building on this overview, the analysis proceeds with a focused examination of each J01 subgroup, highlighting the most commonly used active substances, their economic impact, the comparison with national benchmark and their positioning within the AWaRe profile. To provide perspective on district-level prescribing patterns, hierarchical clustering heatmaps were created for each J01 antibiotic subgroup (Panel D of Figure 4, Figure 5, Figure 6, Figure 7, Figure 8, Figure 9 and Figure 10). In these visualizations, each row depicts an individual molecule, while each column corresponds to one of the 13 health districts. The dendrograms on the top and left sides of each heatmap show the hierarchical relationships, clustering districts and molecules with similar profiles based on three combined metrics: prescription volume, gross expenditure, and the percentage variation (Δ%) in DDD/1000 inhabitants/day compared to the national benchmark. The color gradient illustrates relative deviations from the mean: dark blue shades indicate lower-than-average consumption or negative deviations from the national benchmark, while intense red shades show higher-than-average consumption or significant positive deviations. By analyzing the horizontal blocks and the vertical clustering trees, it is possible to immediately distinguish ‘high-prescribing clusters’ (predominantly red blocks), where certain districts consistently exceed national consumption standards, contrasted with local prescribing patterns that stay controlled or below reference thresholds (predominantly blue blocks). This approach allows a combined overview and granular reading of each therapeutic compartment, helping identify intervention priorities and areas for improved prescribing appropriateness. For analytical purposes, each healthcare district in ASL Salerno was randomly assigned an alphabetical identifier (DS-A to DS-M).

### 2.1. J01A (Tetracyclines)

The J01A subgroup includes tetracyclines [11,12]. In this group, doxycycline (J01AA02) is classified under the WHO AWaRe framework as an Access antibiotic, whereas limecycline (J01AA04) and minocycline (J01AA08) are classified under the Watch group. In 2024, across the thirteen districts analyzed, prescriptions for J01A antibiotics accounted for 0.95% of all J01 prescriptions and 0.66% of gross expenditure. Among tetracyclines, doxycycline represented 40.35% of prescriptions but only 24.69% of gross expenditure. Conversely, limecycline and minocycline together accounted for 59.65% of use and over 75% of total costs, suggesting a relatively higher expenditure for Watch antibiotics (Figure 4A,B).

From the analysis of DDD 1000 ab/die using national reference values, a generalized tendency toward lower doxycycline consumption across most districts is observed, reflecting greater use of Watch molecules (Figure 4C). This pattern was particularly evident in DS-I, where doxycycline use (26.58%) remained markedly below the national benchmark, while both limecycline and minocycline showed substantially higher values. A more balanced distribution is confirmed in DS-E, DS-K, and DS-L, where lower consumption of Watch molecules and relatively higher doxycycline use (53.80%, 51.72%, and 50.0%, respectively) suggested greater adherence to stewardship-oriented prescribing patterns. Although doxycycline use was relatively high, it remained below expectations given its clinical efficacy and cost-effectiveness. In contrast, the preference for limecycline and minocycline could be a representative indication of prescribing practices that are not fully aligned with stewardship goals (Figure 4D).

### 2.2. J01C (Penicillins)

The J01C class includes systemic-use penicillins [13,14]. Within this class, amoxicillin (J01CA04), categorized as Access, represents a widely used antibiotic. The combination of this molecule with clavulanic acid (J01CR02) is also classified as Access. However, it should be used more selectively than monotherapy to avoid unnecessary reliance on broad-spectrum combinations when not strictly required. Instead, piperacillin combined with a beta-lactamase inhibitor (J01CR05) is categorized as Watch. Analysis of 2024 data across the thirteen districts of ASL Salerno shows an overall alignment with antimicrobial stewardship principles. J01C penicillins accounted for 36.51% of total prescriptions and 29.87% of total expenditure. The most frequently prescribed antibiotic was the amoxicillin-clavulanic acid combination (J01CR02), which alone accounted for about 78% of the total J01C prescription volume and absorbed 86% of the expenditure. Although classified as an Access antibiotic, its use should be lower than that of plain amoxicillin, which is less frequently used (9.1% of total prescriptions) despite its favorable profile for many uncomplicated infections. Particularly noteworthy is the use of piperacillin-beta-lactamase inhibitor (J01CR05), which accounted for 3.20% of prescriptions and approximately 10.4% of total spending (Figure 5A,B). Comparison with Italian reference values confirms consistent underutilization of plain amoxicillin compared with the amoxicillin-clavulanic acid combination. At the same time, piperacillin-beta-lactamase inhibitor displayed markedly elevated values in selected districts, especially DS-C, DS-I, and DS-L (Figure 5C). At the territorial level, the distribution appears relatively homogeneous, with average proportions above 97% for Access drugs. Although the overall contribution of antibiotics within the J01C class remains relatively limited, these results suggest that broader-spectrum agents are often preferred (Figure 5D). At the same time, the interpretation of deviations related to piperacillin should be approached with caution, as absolute regional consumption remained low (Italy DDD/1000 ab/die: 0.008; DS-I DDD/1000 ab/die: 0.043) and modest variations could amplify percentage differences. However, in some districts, the expenditure levels associated with Watch drugs exceed thresholds that may warrant closer monitoring. For example, in district DS-C, the percentage of spending on Watch drugs reached nearly 18%, the highest among the thirteen districts, while in DS-L, the value was slightly above 15%. Although Access drug use remains predominant in absolute terms, the economic weight of the Watch antibiotic highlights a trend possibly reflecting prescribing habits requiring further scrutiny.

### 2.3. J01D (Cephalosporins and Other Beta-Lactam Antibiotics)

The J01D class comprises cephalosporins and other systemic beta-lactam antibiotics, Cephalosporins are subdivided into several generations, each characterized by distinct microbiological and pharmacokinetic profiles [15]. First-generation molecules, are classified as Access according to WHO’s AWaRe system and are recommended for many common infections. By contrast, most second-, third-, and fourth-generation cephalosporins, including cefixime (J01DD08), ceftriaxone (J01DD04), and cefditoren (J01DD16), are categorized as Watch. Analysis of 2024 data from ASL Salerno reveals a picture heavily based on Watch molecules. Prescriptions for J01D accounted for 20.94% of the total, absorbing 33.79% of expenditure. Most notably, there was a total absence of Access molecules among the most frequently prescribed agents. Indeed, the three most prescribed cephalosporins, ceftriaxone (J01DD04), cefixime (J01DD08), and cefditoren (J01DD16) (Figure 6A,B), all belong to the Watch category. Among them, cefixime was by far the most used molecule (46.09%). Ceftriaxone accounted for nearly half of total expenditure in the J01D class (47.01%), and cefditoren accounted for 6.69% of J01D prescriptions, with a spending impact of 12.85%. Despite the availability of Access molecules such as cephalexin and cefazolin, these accounted for less than 1.5% of prescriptions and less than 1% of total expenditure. Comparison with national benchmarks confirmed the extensive use of Watch cephalosporins across all districts. Ceftriaxone and cefixime showed consistently higher consumption compared to Italian reference values in most territories, indicating a consolidated preference for broad-spectrum cephalosporins in community prescribing (Figure 6C,D). At the district level, the pattern was uniformly concerning: across all thirteen districts, Access drug use and expenditure remained negligible. On average, over 99% of cephalosporin expenditure was attributable to Watch drugs, with only minor variation across territories. Even in districts with slightly higher Access use, such as DS-H (1.12% of expenditure) and DS-L (0.83%), the impact remained marginal. From these results, a limited use of first-generation cephalosporins and a heavy use of later-generation agents emerge, despite their greater ecological impact on antimicrobial resistance.

### 2.4. J01F (Macrolides, Lincosamides, and Streptogramins)

The J01F class comprises macrolides, lincosamides, and streptogramins [16,17]. Within this class, clindamycin (J01FF01) represents the only active substance classified in the Access category. All other agents fall within the Watch category. Analysis of 2024 data from the thirteen districts of the ASL Salerno highlights an overwhelming predominance of Watch antibiotics, both in terms of prescription volume, expenditure, and DDD/1000 ab/die, with clindamycin being almost entirely marginalized. In fact, in the J01F class, only 0.53% of prescriptions were attributable to clindamycin, whereas more than 99% were accounted for by macrolides and other lincosamides belonging to the Watch category. This imbalance is also reflected in economic terms, as less than 0.5% of expenditure was allocated to clindamycin. Azithromycin emerged as the most frequently prescribed antibiotic, accounting for 53.97% of prescriptions and 49.83% of total expenditure, nearly half of the entire class consumption (Figure 7A,B). This was followed by clarithromycin (39.31%) and roxithromycin (1.24%), while spiramycin, although less commonly used, maintained a stable share of 1.20%. Lincomycin accounted for 7.5% of prescriptions, with a 4.12% impact on expenditure. The comparison with Italian reference values further emphasized the predominance of Watch antibiotics within the J01F class. Consumption of clarithromycin and azithromycin was generally higher than national benchmarks across most districts, confirming the extensive use of macrolides throughout the ASL (Figure 7C). From a territorial perspective, all districts of the ASL displayed a highly homogeneous profile: the percentage of Access utilization ranged from 0.12% (DS-E) to 1.09% (DS-M), while, in terms of expenditure, no district exceeded 1.12% for clindamycin. The highest values of DDD/1000 ab/die were observed in DS-I, both for clarithromycin and in particular for lincomycin. Elevated lincomycin consumption was also detected in DS-D, DS-E, and DS-H (Figure 7D). Although part of the variability observed for lincomycin may reflect relatively low absolute consumption values (Italy: DDD/1000 ab/die: 0.013; DS-I: DDD/1000 ab/die: 0.075), the overall pattern suggests a stable prescribing trend toward broad-spectrum macrolides and lincosamides.

### 2.5. J01G (Aminoglycosides)

The J01G class comprises aminoglycosides [18,19]. Within the WHO AWaRe classification, only amikacin (J01GB06) is listed as an Access agent, whereas tobramycin (J01GB01), gentamicin (J01GB03), and netilmicin (J01GB07) are considered Watch antibiotics. In 2024, within the ASL Salerno, aminoglycosides accounted for 0.51% of total prescriptions and 1.05% of overall antibiotic expenditure. In this context, amikacin represented nearly one quarter of total prescriptions (24%), while Watch antibiotics, led by netilmicin, accounted for the remaining 76%. From an economic perspective, expenditure on Access drugs amounted to approximately 28% of the total, compared with 72% for Watch agents. In the group, netilmicin (J01GB07) was by far the most frequently prescribed and dispensed drug, accounting for about 69% of prescriptions and 66% of costs, followed by amikacin (23.99% and 28.30%) and tobramycin (7.14% and 5.87%) (Figure 8A,B). Comparison with Italian reference values showed widespread use of netilmicin across all districts, with consistently positive deviations throughout the territory. Amikacin also displayed higher consumption than the national benchmark in most districts, whereas tobramycin showed a more variable distribution (Figure 8C). Nonetheless, significant differences emerge between districts. DS-L stands out for its more favorable performance: over 49% of prescriptions and more than 61% of expenditure within the class were attributable to amikacin, in sharp contrast with the average. Similarly, districts DS-C and DS-E showed a more balanced profile, with Access values above 36% in volume and over 47% in expenditure. Conversely, districts DS-F and DS-I were positioned at the opposite extreme, with Access rates below 16%, almost entirely in favor of netilmicin (Figure 8D). Although some of the variability observed in netilmicin use may reflect relatively low absolute consumption values (Italy: DDD/1000 ab/die: 0.003; DS-I: DDD/1000 ab/die: 0.046), the overall pattern suggests a stable prescribing trend towards Watch molecules. The data reveal a prescribing pattern strongly oriented toward netilmicin; this trend appears consolidated, even where lower-resistance-impact alternatives, such as amikacin, are available.

### 2.6. J01M (Fluoroquinolones)

The J01M group includes fluoroquinolones, a class of broad-spectrum antibiotics [20,21]. known for their high potential resistance selection and their association with serious adverse effects [22,23]. According to the WHO AWaRe classification, all systemically active fluoroquinolones are classified as Watch. In 2024, across the ASL Salerno, fluoroquinolone consumption remained substantial, accounting for 13.81% of total prescriptions and 11.96% of overall antibiotic expenditure. The analysis revealed a predominance of three active agents: ciprofloxacin (J01MA02), levofloxacin (J01MA12), and prulifloxacin (J01MA17); which together comprised more than 98% of prescriptions within the group (Figure 9A,B). Specifically, ciprofloxacin accounted for 58.01% of prescriptions and 63.10% of expenditure, followed by levofloxacin with 38.46% of prescriptions and 30.14% of expenditure. Although prulifloxacin had a much smaller prescribing share (2.41%), it accounted for 5.30% of total class expenditure. Other agents, such as moxifloxacin (J01MA14), norfloxacin (J01MA06), lomefloxacin (J01MA07), and rufloxacin (J01MA10), showed marginal values in both prescriptions and economic impact. Comparison with national reference values confirmed the extensive use of fluoroquinolones across all districts. Ciprofloxacin and levofloxacin consumption remained consistently above national benchmarks, indicating a stable reliance on this class of antibiotics in territorial prescribing (Figure 9C,D). Despite their classification in the Watch category, fluoroquinolones continue to be widely prescribed, accounting, as noted above, for approximately 14% of total prescriptions and 12% of total antibiotic expenditure in ASL Salerno in 2024. The near-exclusive use of Watch molecules underscores a structural weakness: there is, in effect, no real modulation of prescribing risk within this class. This evidence reinforces the need for particularly cautious use of fluoroquinolones, limiting their prescription to cases where the expected benefit clearly outweighs both microbiological and toxicological risks.

### 2.7. J01X (Other Antibiotics)

The J01X group encompasses a heterogeneous set of systemic antibiotics that cannot be classified within the main groups. This group includes agents from all three AWaRe categories, reflecting a wide spectrum of indications [24,25,26]. In 2024, antibiotics in the J01X group accounted for 6.75% of total prescriptions and 7.11% of overall antibiotic expenditure. Fosfomycin (J01XX01), classified as a Watch agent, accounted for more than 90% of consumption, as confirmed by comparison with Italian reference values (Figure 10A–C). At the district level, distribution patterns appeared relatively homogeneous: no district showed signs of limiting the use of this Watch antibiotic, with percentages consistently above 90% of total Watch consumption. Among Access molecules, nitrofurantoin (J01XE01) accounted for around 7–11% of consumption in some districts. Higher utilization was observed in districts such as DS-L and DS-C, whereas DS-B, DS-E, and DS-M remained below the national benchmark (Figure 10D). While encouraging, these values remain insufficient relative to their potential as a substitute for fosfomycin. Metronidazole (J01XD01), by contrast, was negligible across all districts. In the Watch category, teicoplanin (J01XA02) stood out for its high economic impact: although it represented only 1.49% of prescriptions, it accounted for 17.20% of expenditure within the J01X subgroup. Despite its limited absolute consumption, several districts showed markedly higher values compared with the Italian reference, particularly DS-I, DS-F, DS-D, and DS-B (Figure 10C,D). This raises important concerns, considering that teicoplanin is primarily indicated for hospital use. The Reserve agent linezolid was detected in small quantities, with distribution across most districts. While volumes remained limited, its territorial presence likely reflects continuation therapy in patients discharged from the hospital and managed through home care services (e.g., integrated home assistance), rather than routine community prescribing. Nonetheless, its diffusion warrants monitoring to ensure appropriate indication and oversight of stewardship.

## 3. Discussion

The analysis of antibiotic prescribing data from ASL Salerno for 2024 reveals a complex system shaped by clinical needs and prescribing habits. What emerges is not merely a quantitative snapshot of consumption but rather a dynamic and heterogeneous representation of how antibiotics are used across different territorial contexts, with sometimes marked differences between districts. This landscape reflects both the weight of clinical demand and the persistence of entrenched therapeutic practices. One of the most striking findings concerns the distribution of consumption according to the WHO AWaRe classification. The predominance of Watch antibiotics over Access agents, evident for most of the analyzed classes, warrants careful consideration. While this may in part reflect specific therapeutic indications (such as in certain complicated infections), its systematic nature suggests a prescribing trend that favors broad-spectrum molecules even where not strictly necessary. In many districts, Watch antibiotics account for 90% or more of prescriptions, while Access drugs, the very agents the WHO recommends prioritizing for their more favorable resistance-risk profile, remain marginal, often confined to less than 10%. While this imbalance raises concerns from a stewardship perspective, the absence of systematic linkage with validated diagnostic codes prevents a definitive assessment of indication-specific appropriateness. Therefore, part of the observed distribution may reflect clinically justified prescribing in specific contexts. The differences observed across districts suggest the possible influence of contextual factors, such as entrenched practices, cultural dynamics, or varying levels of awareness of antimicrobial resistance. Another key issue concerns the relationship between prescribing volume, economic impact, and a strong deviation from the national reference. Some classes, such as cephalosporins, account for a smaller proportion of prescriptions compared with others (e.g., penicillins) but generate higher overall expenditure. This aspect must not be overlooked, as controlling healthcare costs also requires rationalizing the use of more expensive molecules when not strictly necessary. Similarly, the relatively small share of Reserve antibiotics confirms a generally cautious use. In our setting, the detection of linezolid in community pharmacies likely reflects therapeutic continuity for patients receiving home-based care after hospital discharge. Although this pattern does not necessarily indicate inappropriate prescribing, ongoing monitoring remains essential to prevent the gradual shift of Reserve agents into routine outpatient use. It should also be emphasized that certain Access agents, which could be employed much more extensively, are underutilized in favor of Watch antibiotics. In addition to the overall AWaRe distribution, specific stewardship indicators recommended at the national and international level deserve focused consideration. In our analysis, the WHO target of at least 65% Access antibiotics was not achieved. Moreover, the amoxicillin-to-amoxicillin-clavulanate ratio did not align with recommended benchmarks, favoring the use of plain amoxicillin when clinically appropriate. Similarly, the use of second-generation injectable cephalosporins remained relevant despite stewardship recommendations advocating for restricted use. Such prescribing behavior, beyond fostering resistance development, may expose patients to avoidable side effects and unnecessarily increase costs. Despite its strengths, this study has some limitations that should be acknowledged. The primary limitation is the lack of a systematic linkage between antibiotic prescriptions and validated diagnostic codes. While the system technically supports these codes (ICD-9-CM), a preliminary quality assessment revealed significant data gaps, including missing, inconsistent, or duplicated entries. Consequently, we chose to exclude diagnostic data from the final analysis to avoid selection bias that would have arisen from focusing only on prescriptions with valid codes. By prioritizing the complete dataset of over 1 million prescriptions, we ensured a representative overview of territorial consumption and comparison with the national reference. Furthermore, the application of the WHO AWaRe framework provides a robust, internationally recognized proxy for prescribing quality, effectively mitigating the absence of patient-specific clinical indications. The quantitative approach, when integrated with district-level qualitative analysis, indicates that genuine opportunities for improvement exist. Some areas already demonstrate greater adherence to appropriateness principles, with higher reliance on Access antibiotics and a smaller burden of Watch agents. These virtuous models should be valued and leveraged as a foundation for developing corporate-level training programs and shared stewardship strategies. The solution is not a punitive or rigidly prescriptive approach, but rather one that builds awareness, fosters a culture of rational use, and equips professionals with updated tools, clear guidelines, and ongoing clinical support. Ultimately, antibiotic governance cannot be addressed solely as a matter of numbers; it must become an integral component of a systemic vision for community healthcare. Intervening in antibiotic prescribing involves simultaneously addressing professional training, organizational structures, patient communication, and systematic data monitoring. At present, ASL Salerno has defined specific indicators for the J01 class, monitored on a quarterly, semiannual, and annual basis, to evaluate prescribing appropriateness. The analysis is carried out at the district and individual prescriber levels. Therefore, cases of potential inappropriateness are subsequently discussed within the relevant UCAD (District Activities Coordination Office) committees. Data are compared against national and regional benchmarks and compiled into periodic reports that track trends over time and identify prescribing patterns with room for improvement, which are then reviewed with prescribers. In this way, the ASL has established a structured information platform that can support a pathway to transform collected evidence into lasting cultural and organizational change.

## 4. Materials and Methods

### 4.1. Data Collection

Prescription and dispensing data were extracted from the institutional pharmaceutical administrative database of ASL Salerno, implemented via the GecorWeb platform (Campione Informatica S.r.l., Italy), which is used to monitor and manage outpatient pharmaceutical prescriptions and related expenditures within the conventional reimbursement framework. The database includes all National Health Service (NHS)-reimbursed prescriptions dispensed by community pharmacies. It provides aggregated information on ATC classification, active substances, number of prescriptions, gross pharmaceutical expenditure, and the healthcare district of dispensing. All data were extracted in anonymized and aggregated form, in compliance with national data protection regulations and institutional governance policies, and no individual patient- or prescriber-identifiable information was accessed. While the system technically allows for the recording of ICD-9-CM (International Classification of Diseases, 9th Revision, Clinical Modification) diagnostic codes, a preliminary quality assessment revealed significant data gaps. Specifically, the presence of missing, inconsistent, or repeatedly duplicated codes compromised their reliability for robust analytical stratification. To avoid selection bias arising from analyzing only prescriptions with valid diagnostic codes and to ensure the integrity of the findings, the study focused exclusively on consumption patterns, expenditures, and AWaRe distribution. Consequently, a formal assessment of indication-specific appropriateness was excluded from the current analysis.

### 4.2. Data Classification

Antibiotics were classified according to the World Health Organization AWaRe framework, which categorizes agents into Access, Watch, and Reserve groups based on their therapeutic relevance, resistance potential, and priority for antimicrobial stewardship. The AWaRe categorization was applied in accordance with the most recent WHO recommendations available at the time of analysis. Prescribing patterns were examined at both the ATC subgroup and individual active substance levels to capture differences within and across AWaRe categories. The primary outcomes of interest were the number of reimbursed antibiotic prescriptions and their relative distribution across ATC classes and AWaRe categories, as well as the gross pharmaceutical expenditure per group. Secondary analyses focused on variability in prescribing patterns across healthcare districts, with particular attention to differences in the relative use of Access and Watch antibiotics and their associated economic impact.

### 4.3. Data Representations

The extracted data were exported to a tabular format and processed in Microsoft Excel. Data handling procedures included verification of completeness and internal consistency; aggregation of prescription counts and expenditure by ATC class, active substance, AWaRe category, and healthcare district; and calculation of relative percentages to facilitate comparison across districts and antibiotic classes. Descriptive analyses were conducted to characterize antibiotic utilization and expenditure patterns, and results were summarized using tables and graphical representations, such as bar charts, to facilitate visualization of differences in prescription volume and cost distribution among AWaRe categories and across territorial areas.

### 4.4. Statistical Analysis

Quantitative prescription data, consumption metrics, and percentage deviations (Δ%) in DDD/1000 inhabitants/day relative to the national reference baseline were analyzed using MetaboAnalyst 6.0 (http://www.metaboanalyst.ca (accessed on 22 May 2026)) [27,28]. To complement the descriptive bar charts and provide a comprehensive visualization of prescribing patterns, hierarchical clustering heatmaps were generated. The heatmaps were constructed using normalized data matrices, with group clustering based on Euclidean distance and the average linkage method.

## 5. Conclusions

The in-depth analysis of antibiotic consumption in ASL Salerno in 2024 provides a comprehensive picture that is useful for guiding policies to promote the rational use of medications. The observed distribution, characterized by a substantial share of Watch-group molecules, highlights the importance of further stewardship-oriented analyses, ideally integrating diagnostic information, to better distinguish between clinically justified prescribing and areas requiring corrective intervention. It is essential to systematically encourage the use of Access-group antibiotics whenever clinically appropriate, in accordance with WHO-AWaRe principles and national guidelines. Inter-district variability has highlighted opportunities for local improvement, as well as best practices that should be recognized and disseminated. Monitoring activities must therefore continue systematically and in a structured manner, with the goal not only of controlling pharmaceutical expenditure but, above all, of combating antibiotic resistance through targeted and selective use of broad-spectrum agents. Ultimately, the route outlined in this analysis provides a solid foundation for developing local antibiotic stewardship strategies, laying the groundwork for a safer, more effective, and more sustainable use of antibiotics in the community.

## Data Availability

The original contributions presented in this study are included in the article. Further inquiries can be directed to the corresponding author(s).

## Abbreviations

The following abbreviations are used in this manuscript:

ASL	Local Health Authority
WHO	World Health Organization
ATC	Anatomical Therapeutic Chemical
AMR	Antimicrobial resistance
DDD	Defined Daily Dose
DS	Healthcare Districts
NHS	National Health Service
ICD-9-CM	International Classification of Diseases, 9th Revision, Clinical Modification
UCAD	District Activities Coordination Office
